# Intraperitoneal bilirubin administration decreases infarct area in a rat coronary ischemia/reperfusion model

**DOI:** 10.3389/fphys.2014.00053

**Published:** 2014-02-18

**Authors:** Ron Ben-Amotz, John Bonagura, Murugesan Velayutham, Robert Hamlin, Patrick Burns, Christopher Adin

**Affiliations:** ^1^Department of Veterinary Clinical Sciences, The Ohio State UniversityColumbus, OH, USA; ^2^Department of Anesthesiology, Center for Biomedical EPR Spectroscopy and Imaging, Davis Heart and Lung Research Institute, The Ohio State UniversityColumbus, OH, USA; ^3^Sciences Cliniques, Université de MontréalMontréal, QC, Canada

**Keywords:** bilirubin, cardiac ischemia, ischemia reperfusion injury, myocardial infarction, infarct area, fractional shortening, antioxidant

## Abstract

Bilirubin was previously considered a toxin byproduct of heme catabolism. However, a mounting body of evidence suggests that at physiological doses, bilirubin is a powerful antioxidant and anti-atherosclerotic agent. Recent clinical studies have shown that human beings with genetically-induced hyperbilirubinemia (Gilbert Syndrome) are protected against coronary heart disease. The purpose of this study was to investigate whether administration of exogenous bilirubin to normal rats would convey similar protective effects in an experimental model of coronary ischemia. We hypothesized that intraperitoneal bilirubin administration 1 h before injury would decrease infarct area and preserve left ventricular (LV) systolic function when compared to non-treated rats. Coronary ischemia was induced by temporary (30 min) ligation of the left anterior descending coronary artery in control or bilirubin treated rats, followed by a 1-h period of reperfusion. LV function was estimated by measurements of fractional shortening (FS) and fractional area shortening using echocardiography. LV function decreased in both experimental groups after ischemia and reperfusion, although in bilirubin-treated rats FS was less depressed during the period of ischemia (18.8 vs. 25.8%, *p* = 0.034). Infarct size was significantly reduced in the bilirubin treated group compared to the non-treated group (13.34 vs. 25.5%, *p* = 0.0067). Based on the results of this study, bilirubin supplementation appears to provide significant decrease in infarct size although protective effects on LV function were noted only during the period of ischemia. This result also suggests that lipid soluble antioxidant bilirubin prevents the oxidation of cardiolipin and decreases the infarct size in the heart during ischemia.

## Introduction

Heme oxygenase 1 (HO-1), originally known as heat shock protein 32, is the sole enzyme controlling degradation of the heme molecule, forming equimolar quantities of carbon monoxide (CO), biliverdin (BV) and iron (Fe^+2^). BV is then converted into bilirubin by the enzymatic activity of bilirubin reductase (Maines, 1988). Initially, the HO-1 pathway and its byproducts were perceived as steps in a metabolic pathway for catabolism of the toxic heme molecule; however, mounting evidence suggests that HO-1 is an acute phase reactant that plays a crucial role in cytoprotection during cell stress. Upregulation of HO-1 by administration of the enzyme substrate hemin provided remarkable protection against ischemia-reperfusion injury in a rat model of myocardial infarction (Masini et al., 2003). Similarly, transgenic mice overexpressing HO-l in the heart, showed an enhanced functional recovery during reperfusion after ischemia compared with nontransgenic controls (Vulapalli et al., 2002). Evidence now suggests that many of the protective effects of HO-1 are conveyed by the byproducts of heme degradation, bilirubin and CO, and that administration of these molecules may provide a means of supplementing the endogenous protective mechanisms that are already activated in various forms of cardiovascular disease or ischemia-reperfusion injury (Clark et al., 2000; Kato et al., 2003; Nakao et al., 2003; Adin et al., 2005; Nakao et al., 2005, 2006). Bilirubin, in particular, has been tied to cardiovascular disease risk, with numerous studies documenting that serum bilirubin levels are inversely proportional to risk of cardiovascular disease and atherosclerosis in human beings (Schwertner et al., 1994; Breimer et al., 1995; Hopkins et al., 1996; Mayer, 2000; Djousse et al., 2001; Temme et al., 2001; Ollinger et al., 2007; Ghem et al., 2010; Gul et al., 2013). While bilirubin may have several potential mechanisms of cytoprotective action, its primary role is as an antioxidant; bilirubin has been shown to be the most powerful antioxidant in serum, with increasing potency at lower oxygen concentrations (Stocker et al., 1987). At physiologic doses of 2–20 uM, bilirubin has antinitrosative effects (Minetti et al., 1998; Kaur et al., 2003; Kirkby and Adin, 2006) and inhibits vascular smooth muscle proliferation (Ollinger et al., 2005), while at higher doses, cytotoxicity can result (Kapitulnik, 2004).

Although this clinical and experimental evidence supports the importance of bilirubin in preventing chronic vascular disease and atherosclerosis, there have been few investigations of the protective effects of bilirubin in acute myocardial ischemia and reperfusion (IR). Clinical evidence in one recent study does suggest that serum bilirubin levels are inversely proportional to the risk of complications experienced by patients with acute myocardial infarction, but supplementation of bilirubin in these patients has not been investigated (Gul et al., 2013). Based on previous experience in manipulation of the HO-1 pathway in models of acute organ ischemia (Bonnell et al., 2004; Adin et al., 2005; Nakao et al., 2006; Vitek and Ostrow, 2009) and on the importance of free radicals in acute myocardial injury (Werns et al., 1985, 1986) we suspected that pre-treatment with exogenous bilirubin therapy may provide a practical means of reducing tissue injury in the milieu of acute coronary IR injury that occurs during clinical application of interventional cardiology (stenting and balloon angioplasty) or administration of thrombolytic therapies to humans with active myocardial infarction. In this study, we investigated the effects of bilirubin supplementation using a rat model of left anterior descending coronary artery occlusion (LAD) and acute reperfusion injury. We hypothesized that bilirubin administration would decrease infarct area and preserve left ventricular (LV) function when compared to non-treated rats. Our eventual goal is to apply supplemental bilirubin as a prophylactic therapy in human beings at risk for cardiac IR injuries.

## Materials and methods

### Animals

Twelve-week-old, Male Sprague Dawley rats, weighing 220–300 g, were purchased from Harlan Sprague Dawley (Indianapolis, IN). Animals were maintained in a temperature-controlled room and were fed with a standard diet and water *ad libitum*. All procedures were approved by The Ohio State University Institutional Animal Care and Use Committee and were performed in accordance with the Institute for Lab Animal Research Guide for the Care and Use of Laboratory Animals.

### Bilirubin stock solution

Bilirubin stock solutions were prepared to a final concentration of 2 mM. To make the stock solution, 0.0584 g bilirubin (Bilirubin mixed isomers B4126, Sigma-Aldrich, St Louis MO) was dissolved in 0.5 mL 0.2 N NaOH, then total volume was increased by addition of RPMI 1640 + 10% FBS to a total volume of 50 mL. Adjustment of pH to 7.4 was achieved by adding hydrochloric acid. Aliquots of 2 mM bilirubin in RPMI and 10% FBS were stored at −80°C and were protected from light until being thawed and used for each experiment.

### Experimental groups

Male Sprague Dawley rats were randomly assigned into one of three treatment groups. Group 1 was administered the vehicle (PBS; *n* = 5), and Group 2 was administered bilirubin (10 mg/kg; IP; *n* = 5), 1 h before occlusion. To control for effects of anesthesia on cardiac function a third, sham-operated group (*n* = 5) underwent anesthesia and thoracotomy, without LAD occlusion or treatment.

### Surgical procedure

Body weight was recorded for each animal before the procedure. Anesthesia was induced by insufflating 5% isoflurane in 100% oxygen (2–3 min) into an induction chamber. After induction, an injection of 0.4 mL Ketamine (50 mg/mL; 10 mg/kg) was administered intra-peritoneally (IP) to supplement anesthesia during the tracheostomy and to decrease the subsequent requirement for isoflurane. A facemask was then used to deliver 100% O_2_ and isoflurane (1–2%) until tracheostomy access was obtained. A ventral midline cervical approach was made and tracheostomy was performed using a16 gauge intravenous catheter. Each catheter was secured to the skin using 3–0 silk suture, to prevent tube dislodgement during the surgical procedure. After intubation, anesthesia was maintained with positive pressure ventilation (Harvard Rodent Ventilator, Southnatick, MA) using isoflurane (1–2.0%) in 100% oxygen. Oxygen flow rate was set at 0.2 L/min and rats were ventilated at a rate of 95 cycles/min and tidal volume of 2–2.5 mL. A heat lamp was used to maintain body temperature at 37 ± 1°; temperature was monitored continuously using a rectal thermometer (Physitemp™, Clifton, NJ). Each rat was positioned in right lateral recumbency and temporary coronary ischemia was induced as previously described (Masini et al., 2003). Following a left 4th intercostal thoracotomy, the heart was exposed and pericardiectomy was performed. The left auricle was gently retracted exposing the area of the left anterior descending coronary artery. Coronary ischemia was induced (group 1, 2) and maintained for 30 min by encircling the left anterior descending (LAD) branch of the left coronary artery, 1–3 mm from the tip of the normally positioned left auricle, with 6–0 silk suture with a tapered needle (Sofsilk™, Covidien, Norwalk, CT). The suture was tightened over a 2 mm section of PE-50 tubing to prevent vascular injury and to permit the release of the ligature during the reperfusion period. After release of the suture, reperfusion was allowed to occur for 60 min. Reperfusion of the previously occluded coronary artery was confirmed by visual inspection.

### Echocardiography

Left ventricular function was assessed with two-dimensional and M-mode echocardiography (Hagar et al., 1995). Images of the left ventricle (LV) were obtained at three different time points: after exposing the heart but prior to ligation; at the end of the ischemic period; and at the end of the reperfusion period. Before each echocardiographic measurement, saline was infused into the left hemithorax to minimize air artifacts and the hemithorax was temporarily closed using 4–0 Nylon suture (Ethicon, Johnson and Johnson, New Brunswick, New Jersey). Heart function was assessed by echocardiography at three different time points: prior to ischemia (baseline), after 30 min of LAD ligation (end of ischemia), and at 1 h post reperfusion (end of reperfusion) using an ultrasound unit (Vivid7™, GE Medical Systems, Milwaukee, WI, USA) equipped with a 13-MHz linear phase arrayed transducer (GE i13L) and proprietary software for rodent imaging. Acquisition frame rates were 159/s. The heart was first imaged with 2D echocardiography using a short axis-imaging plane at the level of the papillary muscles. The M-mode cursor was then placed perpendicular to the ventricular septum and LV posterior wall to acquire M-mode imaging of the LV. Images were stored in digital format on a magnetic optical disk for subsequent review and analysis. Measurements of the LV internal diameter at end- diastole (LVIDd) were taken at the time of maximal LV diastolic dimension, whereas measurements of the LV internal diameter at end-systole (LVIDs) were taken at the time of the apogee of the systolic excursion of the posterior wall. Five different measurements were taken and the average number was used as a single data point for statistical analysis. Left ventricular fractional shortening (FS) was calculated by the formula: FS (%) = (LVIDd-LVIDs / LVIDd) × 100. Fractional area shortening (FAS) was calculated by tracing the endocardial border at the maximal LV diastolic diameter, and minimal LV systolic diameter. FAS was calculated by the formula FAS (%) = (LVAd-LVAs/LVAd) × 100.

### Tissue sampling

Before termination of the experiment, the LAD was re-occluded and 1mL of 1% Evans blue solution (Sigma-Aldrich, St. Loius, MO) was perfused in a retrograde manner with a 22-gauge needle inserted into the ascending aorta (group 1, 2) to identify the area at risk of infarction. The heart was then removed and placed in a rodent slicer matrix (Zivic instruments, Pittsburgh, PA) and cooled in a −80°C freezer for 10–15 min to facilitate slicing. After freezing, 5–6 heart sections of 2 mm thickness were made. Sections of the ventricles from the level of the ligature to the apex were then incubated in 2% triphenyltetrazolium chloride (TTC; Sigma-Aldrich, St. Louis, MO) solution in a 37°C bath for 20 min to visualize the unstained infarcted region. After TTC staining, viable myocardium stains brick red and the infarct appears pale white. This staining technique relies on the ability of dehydrogenase enzymes in viable myocytes to convert the stain to a brick red color. Heart slices were placed in buffered formalin and infarct size analysis of the third most distal heart slice (from apex-to base) was performed after 24 h.

### Determination of infarct area

Infarct area was identified using tetrazolium chloride as a vital staining technique (Fishbein et al., 1981; Ytrehus et al., 1994). Digital images were made of the apical side of the third slice of the heart from the apex. The area of infarction and LV areas at risk were determined by computerized planimetry using an image analysis software program (Image/J, National Institutes of Health). Area at risk for infarction was defined using Evan's blue dye that was injected during LAD occlusion at the termination of the experiment. Within the area at risk, the infarct area was identified by a pale region in the absence of red TTC staining and was expressed as a percentage (infarct area /area at risk × 100). Measurements were repeated 5 times for each heart and the average of these 5 measurements was used to calculate area at risk and infarct area. The individual conducting the measurements was blinded to the experimental group.

### Statistical analysis

Statistical calculations were performed using a computer software program (Statview, SAS Institute, Inc, Cary, NC). Data were tested for distribution and equality of variance. Data for each time period and treatment group were summarized and are reported as means ± SD. Fractional shortening (FS%) and FAS were compared over the 3 different time periods and between treatment groups, using a two-way ANOVA for repeated measures (with time period as the within-groups and treatment as the between-subjects factors). Comparison of infarct size% between the two treatment groups was made explored using Student's *t*-test for unpaired data. A *p* < 0.05 was considered statistically significant.

## Results

### Bilirubin pretreatment significantly reduced relative infarct size after LAD occlusion and 1 h of reperfusion

**Figure 1:**
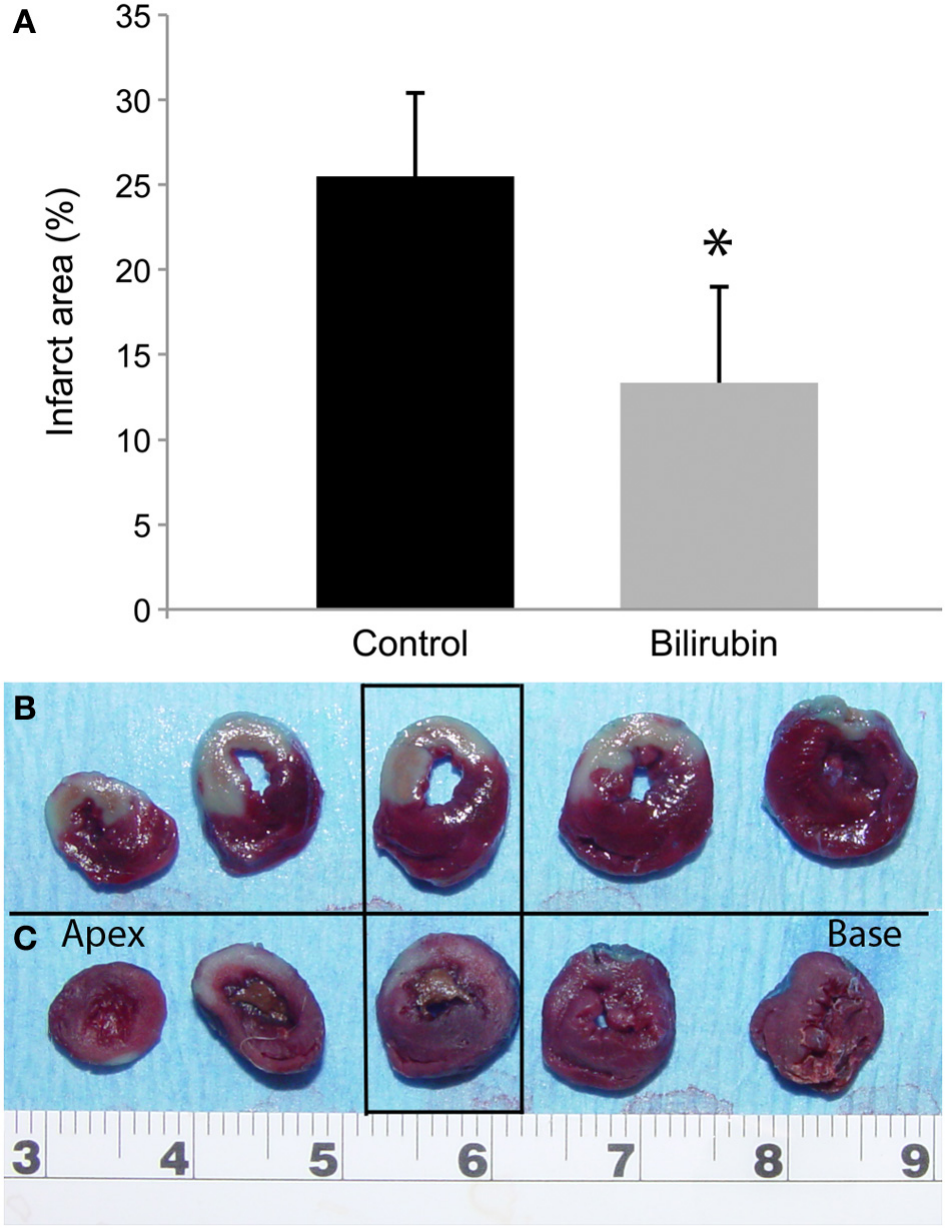
**Bilirubin significantly decreases infarct area after LAD occlusion.** Myocardial infarction associated with 30 min ischemia and 1 h reperfusion was documented using TTC staining. Mean infarct size is reported as a percentage of the area at risk (infarct area %) in the control rats and in rats that received 20 uM/kg IP bilirubin treatment 1 h before ligation of the LAD **(A)**. While there was considerable variation among rats, as a group, infarct area % for bilirubin treated rats was significantly smaller in the bilirubin treated group when compared to the non-treated group (^*^p = 0.0067). Representative image of TTC stained heart slices are arranged from apex (left) to heart base (right) for control **(B)** and bilirubin treated rats **(C)**. Infarct size was quantitated using intravital staining with Triphenyltetrazolium chloride (TCC) in the third heart slice from the apex (in rectangular box). Active dehydrogenases in viable myocardial cells convert the water-soluble compound into a brick-red, insoluble precipitate; in contrast, the infarct area in this image is pale-white. Evan's blue staining was used to identify the area that was unaffected by the ligation, with the remaining area representing the area at risk.

Successful coronary artery occlusion was confirmed in each animal by visual examination of myocardial pallor, by echocardiographic identification of real-time, segmental wall motion abnormalities, and quantitatively by vital staining. Left anterior descending artery occlusion consistently created a large area at risk, as shown by delineation using Evans blue dye (non-ischemic zone) (Figure 1). Staining with TTC delineated the infarct site in the transverse heart section (third section from apex), with the area of infarction appearing pale, and the area at risk in red (Figure 1). Mean infarct size reported as a percentage of the area at risk in the non-treated rats was 25.51% ± 4.9 whereas the mean infarct size in the treated bilirubin group was 13.34% ± 5.7. Infarct size was significantly smaller in the bilirubin treated group when compared to the non-treated group (*p* = 0.0067, Figure 1).

### Bilirubin supplementation had minimal effects on cardiac function in the acute reperfusion period

**Figure 2:**
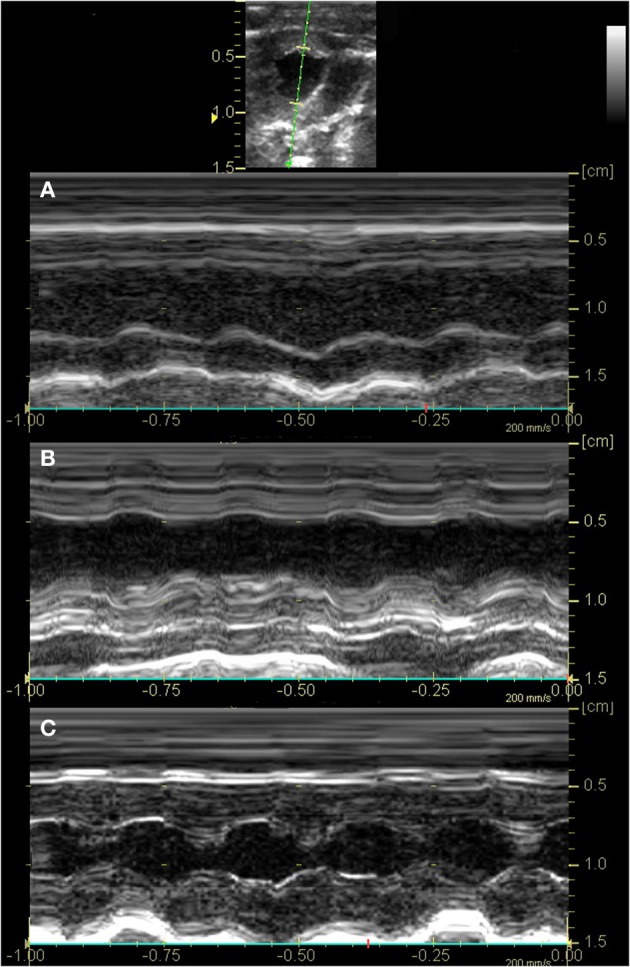
**Fractional shortening was decreased in all groups after LAD occlusion.** Two-dimensional imaging of the LV at the level of the papillary muscles was obtained. The anatomical M-mode cursor (green line) was then placed perpendicular to the ventricular septum and LV posterior wall to acquire M-mode imaging of the LV as indicated in the image (top). Representative images of rats in the control **(A)** and bilirubin treated group **(B)** show segmental hypokinesis secondary to myocardial ischemia and severely reduced systolic function at the time of reperfusion (FS 11 and 21%, respectively), while systolic function was normal for sham rats **(C)** at the equivalent time period (FS 44%).

**Figure 3:**
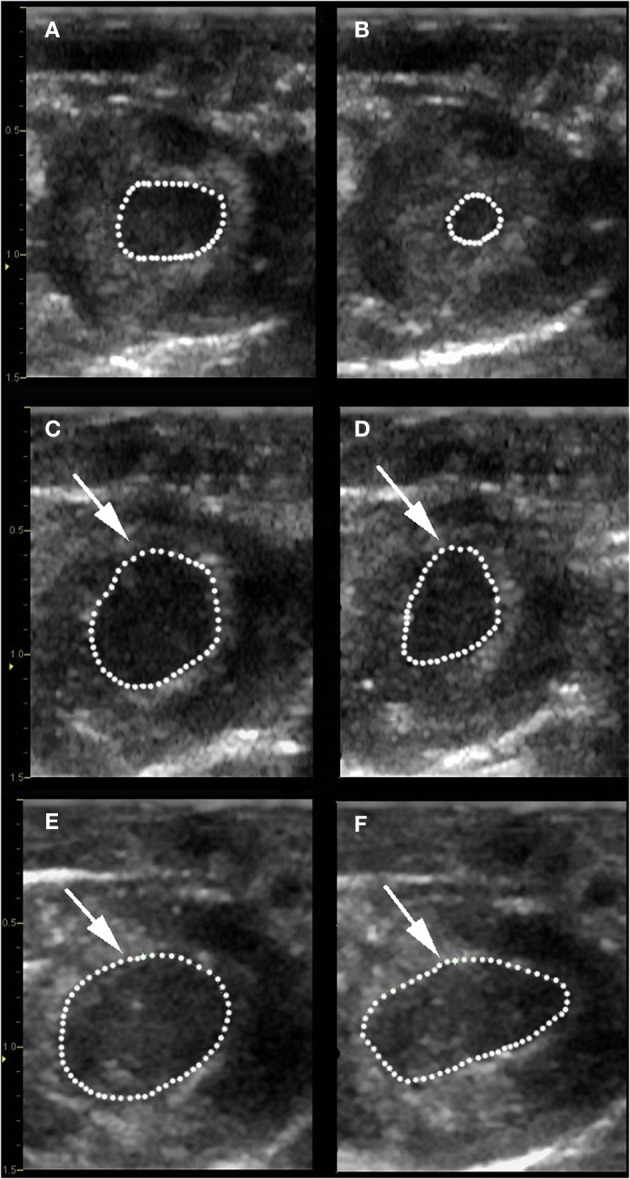
**Determination of area shortening.** Baseline measurements of the LV internal area at end-diastole (LVAd) **(A)** and at end-systole (LVAs) **(B)** were obtained in a control rat prior to ischemia. Fractional area shortening was calculated using the formula: (LVAd-LVAs/LVAd) × 100. Repeat images show alterations in LVAd and LVAs for a control rat **(C,D)** and bilirubin treated rat **(E,F)** after 30 min of ischemia. Note the severely decreased area shortening % that occurred after LAD occlusion and the segmental hypokinesis in the area of myocardial injury (white arrows) in both groups.

**Figure 4:**
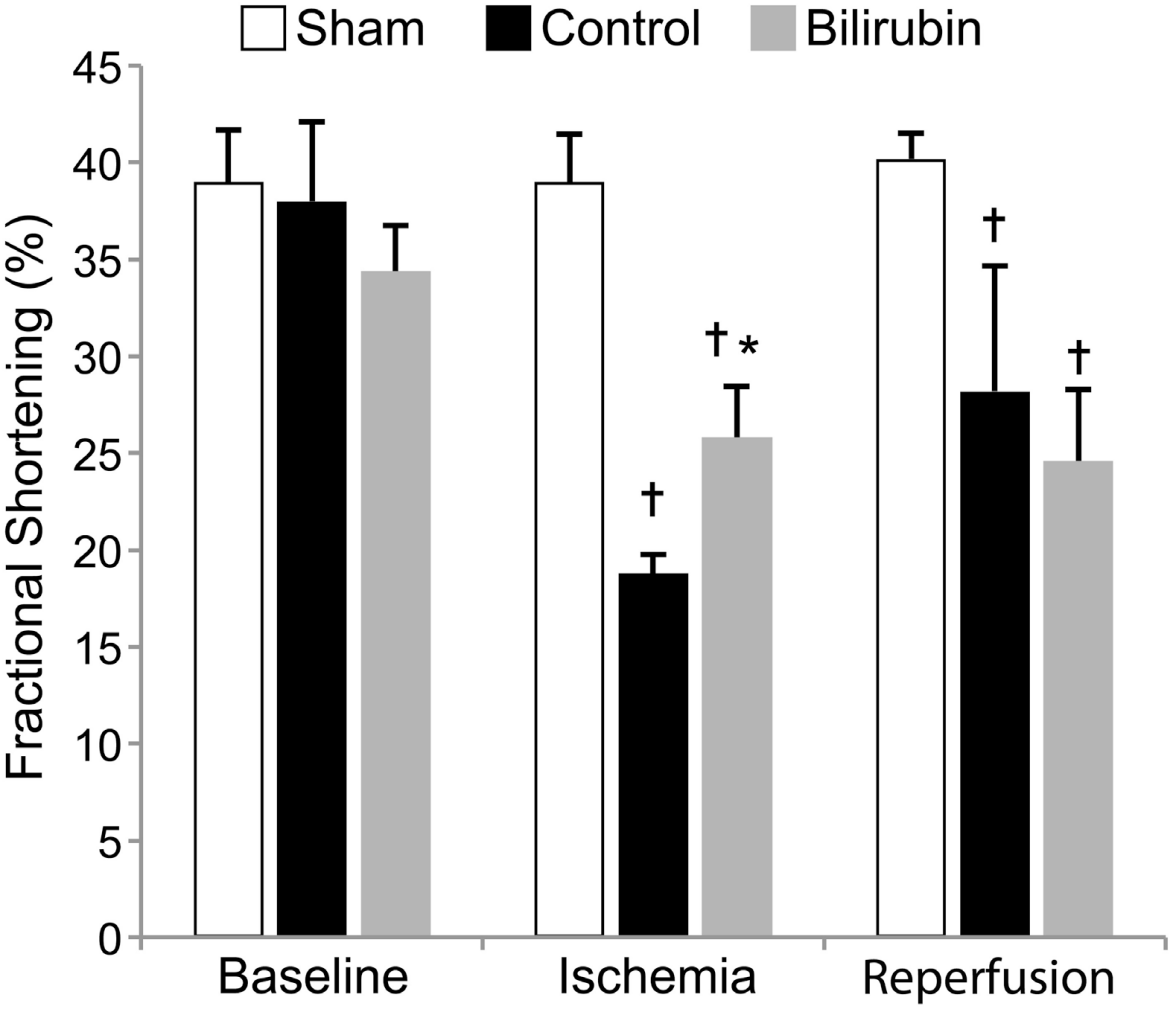
**Bilirubin improves fractional shortening at the end of ischemia.** Mean (s.e.m.) fractional shortening measurements obtained by echocardiography of sham (non-ischemic), control (ischemia + vehicle) and control (ischemia + 20 uM bilirubin IP 1 h before induction) groups at baseline, at the end of 30 min of ischemia and 1 h after reperfusion. Left ventricular function remained relatively constant throughout the anesthetic period in the sham group while ischemia caused significant decreases in fractional shortening in both control and bilirubin treated rats. LV systolic function was higher in the bilirubin-treated group during ischemia, but this effect was not evident after reperfusion. ^*^p < 0.05 vs. control, ^†^*p* < 0.05 vs. sham.

**Figure 5:**
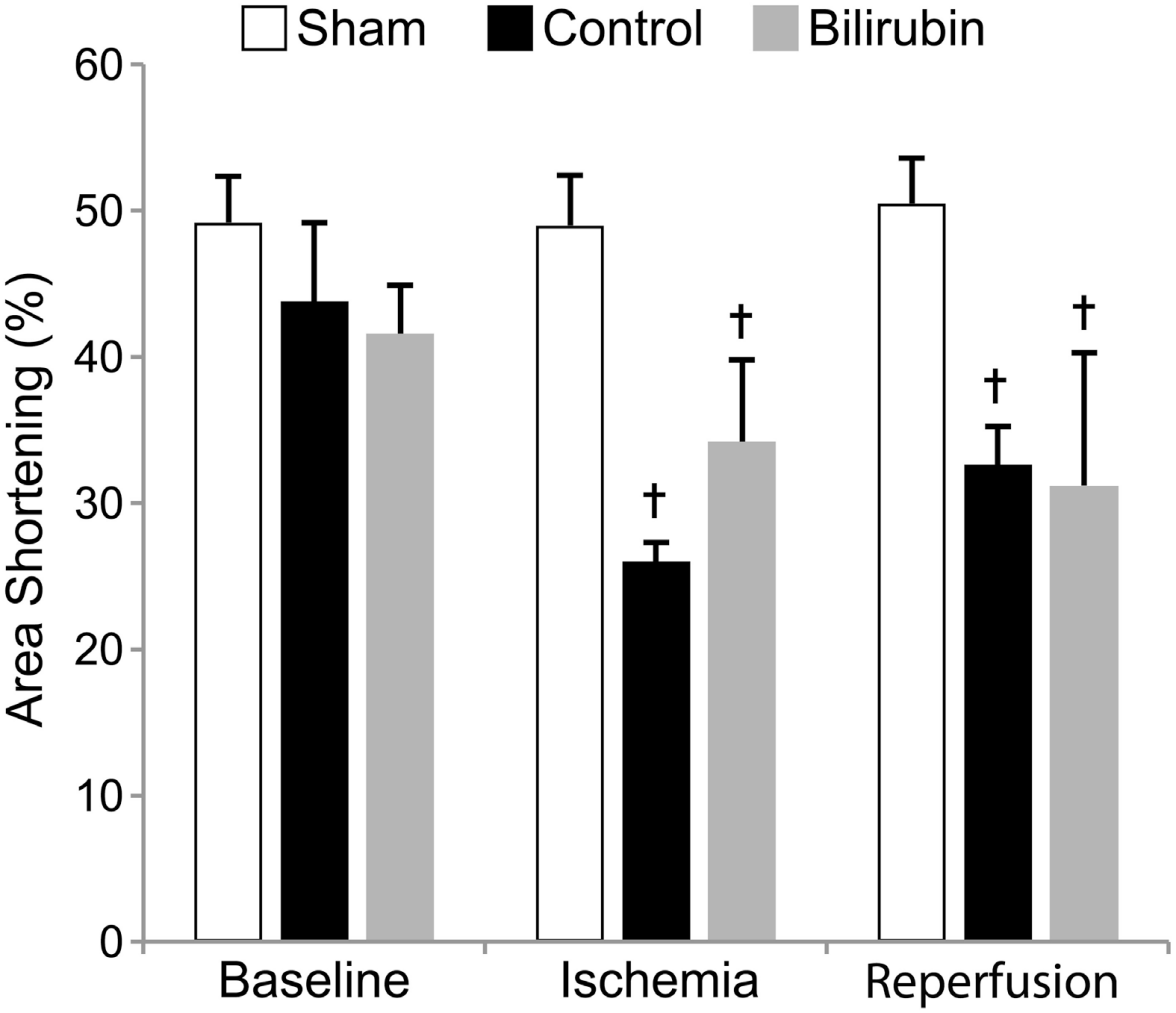
**Bilirubin shows no detectable effect on area shortening.** Fractional area shortening was calculated as an additional measure of global left ventricular function in sham (non-ischemic), control (ischemia + vehicle) and control (ischemia + 20 uM bilirubin IP 1 h before induction) groups at baseline, at the end of 30 min of ischemia and 1 h after reperfusion. Similar to results for fractional shortening, LAD occlusion caused significant decreases in FAS in both control and bilirubin treated rats. Bilirubin treated rats showed a trend toward better LV systolic function at the end of ischemia, but the difference was not statistically significant (*p* = 0.098). ^†^*p* < 0.05 vs. sham.

Left ventricular systolic function was profoundly decreased in both experimental groups after IR, with segmental LV wall dysfunction and resulting decreases in FS% (Figure 2) and AS% (Figure 3). LV function as estimated by FS% was less depressed during the period of ischemia in BR-treated rats, when compared to control (vehicle treated) rats (*p* = 0.034). However, no significant difference in measurements of LV function remained at the time of reperfusion (Figure 4). Left ventricular function was relatively unchanged throughout the anesthetic period in sham rats, validating the model and echocardiographic technique. For FAS, LV function decreased significantly in both experimental groups after IR. Although FAS showed a similar trend toward improvement in the bilirubin group during the time of ischemia (Figure 5), differences from control were not statistically significant (*p* = 0.098). Furthermore, there was no significant difference in measurements of LV, FAS at the time of reperfusion.

## Discussion

Results of this study showed that bilirubin pre-treatment significantly reduced infarct size in a rodent model of acute LAD occlusion and 1 h reperfusion. Mean infarct size was nearly 50% less in the treated group when compared to untreated controls. These results correlate with the protective effect seen after pharmacologic or genetic upregulation of HO-1 prior to tissue injury (Yet et al., 2001; Melo et al., 2002; Tullius et al., 2002; Vulapalli et al., 2002) and suggest that bilirubin therapy may offer a simple pharmacologic means to substitute for the effects of HO-1 upregulation in a safe and readily achievable manner. Although high doses of bilirubin are potentially toxic in neonates (Scheidt et al., 1977; Laws, 1978; Hansen, 1993), the doses used in our current study are designed to produce serum concentrations that are within the physiologic range in humans (5–20 μM; Sies and Stahl, 1995) and would not be expected to incur any risk in a healthy adult. IP administration of bilirubin has been shown to increase plasma levels of bilirubin, reaching a peak at approximately 1 h after administration (Wang et al., 2006). Bilirubin is then quickly eliminated from the circulation and redosing or chemical modification of the molecule would be necessary to create a sustained effect. In type 2 Crigler-Najjar syndrome individuals the level of bilirubin maintain at 19 mg/dL for 50 years without detectable damage to the nervous system (Gollan et al., 1975). Hence, in our study dosages between 3 and 20 mg/kg have been used to increase total serum bilirubin concentrations into the physiologic range (0.5 to 1 mg/dL) and to achieve physiologic effects in rodent models (Wang et al., 2004, 2006; Lanone et al., 2005; Kirkby et al., 2007). Preconditioning therapies are typically most effective when initiated prior to the onset of tissue injury. Although bilirubin had significant protective effects in this model, treatment 1-h before myocardial infarction would be impractical as a clinical treatment of human myocardial infarction. However, in elective situations where ischemia might be predicted, as with an interventional coronary procedure, pretreatment with bilirubin could be a potential option. Future studies will investigate the efficacy of bilirubin administration during ischemia, but prior to reperfusion- a dosing protocol that would be more clinically applicable. Moreover, in the serum, ~99% of bilirubin is bound to plasma protein and hence unavailable for intracellular actions (Sedlak and Snyder, 2004). Alternatively, creation of targeted drugs that can produce more sustained increases in serum bilirubin concentration by releasing it from the binding sites in the serum proteins may facilitate prophylactic treatment of individuals that are at risk of myocardial infarction. Limitations in group size restricted our ability to measure function and infarct size at multiple time points after injury in this initial study. In addition, lack of a control group that received bilirubin alone (without LAD occlusion) makes us unable to discern whether bilirubin has direct effects on LV function in the healthy animal. Based on the positive effects seen in this initial study, future experiments involving additional control groups and recovery models are indicated.

A variety of methods are available for measurement of infarct size, although vital staining techniques are considered the standard method (Fishbein et al., 1981; Ytrehus et al., 1994). TTC is typically used to stain viable tissue where active dehydrogenases in myocardial cells convert the water- soluble compound into a brick-red, insoluble precipitate. Tetrazolium chloride staining is a well-validated method that allows the early detection of myocardial infarction (Fishbein et al., 1981; Ytrehus et al., 1994). Tissue lacking hydrogenase enzyme activity is either dead or destined to die. It should be noted however, that tissue that stains positive is not necessarily healthy and injury may progress for hours or even days after reperfusion. The longer the reperfusion period, the more reliable the staining method becomes for discriminating between dead and viable tissue. While some researchers state that reperfusion times of less than 3 h (2 h for perfused hearts) can make this technique less reliable (Fishbein et al., 1981; Ytrehus et al., 1994), others report that the technique can be used reliably after 1 h of reperfusion (Clark et al., 2000; Masini et al., 2003).

Our results showed minimal differences in echocardiographic function at the time of reperfusion despite a clear decrease in infarct size in the bilirubin pretreated group. The marked decrease in cardiac function seen in both control and bilirubin treated hearts is likely due to a phenomenon termed myocardial stunning. Reversibly damaged myocytes do not contract as efficiently as they did in the control state. Myocardial stunning is a reversible reduction in cardiac function that is not accounted for by tissue damage; rather, ischemia results in conversion to anaerobic glycolysis and lactate accumulation with eventual cessation of contraction in the entire area at risk persists for hours or days. It has been shown that myocytes reperfused late in the reversible phase of ischemia still exhibit stunning 48–72 h after being reperfused and that this phenomenon is due to free radical induced injury (Bolli, 1990, 1992). This reverse phenomenon would not have been encountered in our acute model. In some circumstances, myocardial stunning can be ameliorated if free radical scavengers are given immediately before reperfusion (Bolli, 1988). We elected to use transthoracic echocardiography to estimate systolic LV function. Echocardiographic measurements of FS% and FAS% are well established as methods for estimation of global function. However, both of these indices may be suboptimal for assessing regional wall motion abnormalities typical of focal myocardial ischemia. Both FS and FAS are intended to estimate ejection fraction of a symmetrically contracting ventricle. While FAS did identify wall motion abnormalities in real time, the quantitation of these was likely attenuated by considering contraction of the entire ventricle or the single line method of the M-mode study, which may not effectively represent segmental function. Physicians typically score regional wall motion abnormalities in ischemic patients when evaluating 2D echocardiograms; however, these scores are subjective and at best semiquantitative. We attempted to apply myocardial strain imaging in these studies, but were disappointed by the reproducibility in pilot studies and did not include these methods in the current report. However, with refinements in transducers and algorithms, this approach would seem feasible in future studies. The use of strain might also mitigate the impact of tethering, wherein adjacent segments can affect the motion of each region leading to either over-representation or under-representation of the true segmental wall motion abnormalities. Lastly, ischemia also can affect ventricular diastolic function regionally and globally, but this was not evaluated in these rats.

Due to the invasive nature of this study, all measurements were obtained with the rats under general anesthesia maintained with isoflurane. It is known from previous rodent studies that the dose and type of anesthetic may influence heart rate, LV systolic and diastolic function, LV thickness and LV cavity dimensions. When comparing isoflurane (ISF) and ketamine to pentobarbital used as anesthetic agents, isoflurane and ketamine yielded echocardiographic LV structural and functional data different to those obtained in conscious rats, with isoflurane anesthesia resulting in significantly lower FS and AS% as compared with other anesthetic agents (Plante et al., 2006; Stein et al., 2007; Droogmans et al., 2008; Jakobsen et al., 2010). Although general anesthesia is likely to have affected cardiac functional parameters in our model, measurements of LV function (FS and FAS%) remained constant throughout the anesthetic period in sham-operated rats, validating the model and echocardiographic technique.

In rat, it has been demonstrated that protective effects of bilirubin rinses on liver for tissue grafts by reducing hepatocellular oxidative damages, post-tranplantational reperfusion injuries, and transplanted organ dysfunction (Kato et al., 2003). Our results demonstrate that bilirubin has cardiac protective effect against IR injuries/oxidative damages. However, the protective action of rinse of bilirubin on post-tranplantation reperfusion injury and organ dysfunction in the heart remains to be explored. Our *in vivo* promising results show that there is a potential opportunity for the bilirubin rinses for protection against post-tranplantational reperfusion injury and transplanted graft dysfunction in cardiac tissues. This simple costless procedure could be of greater advantage for the heart transplant.

**Figure 6:**
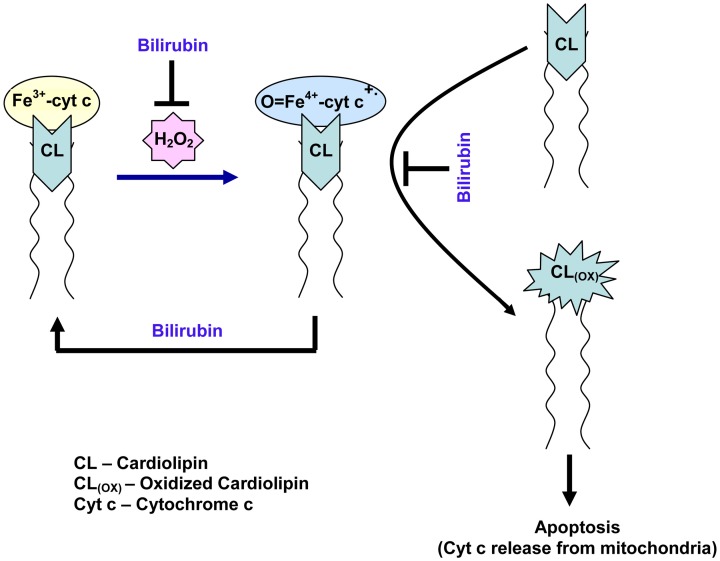
**Proposed Model of lipophilic bilirubin inhibition of peroxidase activity of cyt c, lipid (cardiolipin) oxidation, and apoptosis in cardiomyocytes**.

Based on our studies and reported literature, we propose that antioxidant bilirubin inhibits the peroxidase activity of cytochrome c, oxidation of lipids (cardiolipin), and apoptosis in cardiomyocytes as depicted in Figure 6. It has been reported that oxidative stress or damage is increased in heart disease patients and during surgical reperfusion of the whole heart (Ferrari et al., 1998; Tomaselli and Barth, 2010). Oxidative stress is associated with increased formation of ROS. The level of ROS is increased under ischemia and greatly accelerated with the sudden reintroduction of oxygen at the onset of reperfusion (Zweier and Talukder, 2006). The increased production of ROS leads to oxidative damage of various biomolecules and lipids. In heart, apoptosis increases during IR (Dispersyn and Borgers, 2001; Cheng et al., 2010). Mitochondria are now recognized to play a critical role in mediating both apoptotic and necrotic cell death. In the mitochondrial inner membrane, cardiolipin (CL) is an acidic phospholipid confers fluidity and stability on the membrane (Orrenius and Zhivotovsky, 2005; Gonzalvez and Gottlieb, 2007). Cytochrome c (cyt c) is anchored to the mitochondrial membrane through both hydrophobic and electrostatic interactions with CL and form CL-cyt c complex (Orrenius and Zhivotovsky, 2005; Gonzalvez and Gottlieb, 2007). The release of cyt c from the mitochondria is an early step in apoptosis (Kluck et al., 1997). The peroxidase activity and oxidation of cyt c and CL respectively increases during apoptosis (Velayutham et al., 2011). The oxidized CL facilitates the release of cyt c from mitochondria during apoptosis. Studies have shown that physiological role of bilirubin is an endogenous antioxidant (Sedlak and Snyder, 2004; Sedlak et al., 2009). Bilirubin has cytoprotective effects against reactive oxygen species and lipid radicals (Sedlak et al., 2009). Bilirubin is hydrophobic and its antioxidant effect exceeds that of vitamin E toward lipid peroxidation (Stocker et al., 1987). It is well documented that bilirubin prevents oxidative degradation of lipids and improves cell survival (Sedlak et al., 2009). In men, it has been demonstrated that inverse relationship between serum bilirubin and atherosclerosis (Novotný and Vítek, 2003). In our study the administration of bilirubin decreased the infarct size in the heart during ischemia. This *in vivo* result suggests that lipid soluble bilirubin could prevent the oxidation of lipids such as cardiolipin and regulate apoptotic cell death and decreases infarct size in the heart during IR.

In conclusion, bilirubin supplementation was found to significantly reduce infarct area in an LAD occlusion model of myocardial IR injury. Minimal improvements were noted in LV function in the early reperfusion period. Current evidence suggests that administration of bilirubin may provide an effective means of pre-conditioning prior to elective procedures or graft involving cardiac IR injuries. Conceivably, administration of drugs to release bilirubin from the binding sites in serum proteins is an alternate method of treatment for the protection of individuals that are at risk of myocardial infarction.

### Conflict of interest statement

The authors declare that the research was conducted in the absence of any commercial or financial relationships that could be construed as a potential conflict of interest.
